# Lightweight cattle pose estimation with fusion of reparameterization and an attention mechanism

**DOI:** 10.1371/journal.pone.0306530

**Published:** 2024-08-07

**Authors:** Enming Zhao, Bobo Chen, Hongyi Zhao, Guangyu Liu, Jianbo Jiang, Yanpeng Li, Jilei Zhang, Chuang Luo

**Affiliations:** 1 School of Engineering, Dali University, Dali, China; 2 Institute of Eastern Himalayan Biodiversity Research, Dali University, Dali, China; Universiti Tunku Abdul Rahman, MALAYSIA

## Abstract

Heatmap-based cattle pose estimation methods suffer from high network complexity and low detection speed. Addressing the issue of cattle pose estimation for complex scenarios without heatmaps, an end-to-end, lightweight cattle pose estimation network utilizing a reparameterized network and an attention mechanism is proposed to improve the overall network performance. The EfficientRepBiPAN (Efficient Representation Bi-Directional Progressive Attention Network) module, incorporated into the neck network, adeptly captures target features across various scales while also mitigating model redundancy. Moreover, a 3D parameterless SimAM (Similarity-based Attention Mechanism) attention mechanism is introduced into the backbone to capture richer directional and positional feature information. We constructed 6846 images to evaluate the performance of the model. The experimental results demonstrate that the proposed network outperforms the baseline method with a 4.3% increase in average accuracy at OKS = 0.5 on the test set. The proposed network reduces the number of floating-point computations by 1.0 G and the number of parameters by 0.16 M. Through comparative evaluations with heatmap and regression-based models such as HRNet, HigherHRNet, DEKR, DEKRv2, and YOLOv5-pose, our method improves AP_0.5_ by at least 0.4%, reduces the number of parameters by at least 0.4%, and decreases the amount of computation by at least 1.0 GFLOPs, achieving a harmonious balance between accuracy and efficiency. This method can serve as a theoretical reference for estimating cattle poses in various livestock industries.

## 1. Introduction

Applying computer vision technology in intelligent cattle herd monitoring has garnered considerable scholarly interest, presenting an efficient alternative to the conventional, labor-intensive approach of manual cattle observation and recording individual cattle data. Using computer vision methodologies to analyze cattle images or videos enables the precise identification and location prediction of crucial body parts, including the head, spine, and leg keypoints. The systematic alignment of these keypoints enables meticulous extraction of the cattle skeletal framework [1], which is fundamental for estimating cattle poses. Cattle behavioral patterns are closely correlated with their physiological condition and health status, as evidenced by numerous studies [2–4]. Notably, manifestations of lameness in cattle are frequently accompanied by discernible alterations in spinal height [5]. Additionally, there is a tendency for cattle experiencing illness to exhibit increased recumbency, whereas those in estrus are more inclined toward extended periods of standing. Variations in behavioral patterns serve as a pivotal source of insights for evaluating the health status of cattle populations [6,7]. Behavior is composed of different pose combinations, where pose estimation is a critical component of behavioral analysis. Accurate information on cattle poses is essential for assessing the health status and welfare of captive animals.

In recent years, substantial advancements have been made in pose estimation techniques, which are predominantly facilitated by incorporating deep learning algorithms [8,9]. Of particular note is the approach based on convolutional neural networks, which exhibits robust feature extraction and generalization capabilities. These methods can efficiently and autonomously learn and distill the representational keypoint characteristics from intricate image datasets, markedly diminishing the dependence on manually crafted features [10,11] Currently, the prevalent keypoint detection networks include DeepPose [12] OpenPose [13], HRNet [14], HigherHRNet [15], DEKR [16], DEKRv2 [17], YOLOv5-pose [18], YOLOv7-pose [19] and YOLOv8-pose. Numerous studies have applied deep learning networks to cattle pose estimation and behavioral classification. Li et al. [20] proposed a temporal aggregation network capable of effectively identifying early signs of lameness in cattle through the integration of micromotor features and spatiotemporal analysis. Li et al. [21] achieved precise recognition and estimation of cattle poses utilizing three deep cascading convolutional models. Their study processed 2,134 RGB images of 33 dairy and 30 beef cattle, which were taken under natural conditions on livestock farms. Fan et al. [22] designed the CMBN, a simple multibranch network based on HRNet, attaining an impressive 93.2% AP on the supplementary NWAFU cattle dataset. Russello et al. [23] employed the T-LEAP pose estimation model, an adaptation of the LEAP model into a time series framework, to identify keypoints in image sequences for predicting cattle poses. The model demonstrated proficiency, as evidenced by an average probability of correct keypoint (PCK) of 93.8% on cattle objects. However, the scope of testing was limited, as the efficacy of the model was validated on a single cattle specimen. Gong et al. [24] employed the YOLOv4 model to generate initial detection bounding boxes for cattle. This approach was integrated with a multilevel convolutional neural network designed to extract heatmaps and partial affinity fields (PAFs) of keypoints, which aids in accurately matching these points for individual cattle. Additionally, they implemented a fully connected neural network (FCNN) to distinguish among three typical behaviors of cattle. This approach enabled not only precise pose estimation but also effective behavioral classification in scenarios involving multiple cattle.

However, most of the above methods rely on heatmap analysis and often require tedious post-processing to optimize network performance. In various fields have emerged new ideas for developing neural network. Li et al. [25] developed a method for individual cow recognition using an improved lightweight convolutional neural network based on AlexNet, which employs multi-scale convolution, shortcut connections, an improved Inception module, and attention mechanisms for accurate identification in complex backgrounds. Fu et al. [26] proposed a model combining Null Convolution, Ghost module, and CBAM Attention Mechanism for better key information extraction. Their model achieves 98.58% recognition accuracy in complex backgrounds, reduces parameters by a factor of 24.85, and has a size of only 3.61MB, enabling non-contact cow recognition. Li et al. [27] also proposed LiteBlock, a lightweight basic block for human pose estimation. By using deeply separable convolutional and inverse bottleneck layers, LiteBlock significantly accelerates network computation and reduces parameters. Li et al. [28] developed a human pose estimation model based on the concept of human behavior recognition. The proposed method employs a lightweight multi-scale coordinate attention mechanism. By integrating GhostNet to reduce model redundancy and using the CA attention mechanism to enhance orientation and position sensing, efficient multi-scale feature fusion is achieved through the BiFPN module. Experimental results indicate that this method significantly improves the average accuracy on the COCO 2017 dataset while minimizing network parameters and computational effort, demonstrating superior performance with a lightweight network architecture.

Inspired by the success of lightweight models in human pose estimation, this study draws on the thinking of a lightweight model for human pose estimation and applies it to cattle pose estimation, thereby contributing to the advancement of smart cattle farming. In particular, this paper proposes an end-to-end, lightweight network for cattle pose estimation. This approach is based on YOLOv8-pose, which integrates an attention mechanism with a reparameterized framework. The contributions of this paper are as follows:

1. By integrating three sets of unparameterized, weighted SimAM [29] attentional mechanisms, this approach acquires orientation and location-sensitive feature information. This enhancement significantly bolsters the network’s robustness in tasks involving complex backgrounds or occlusion scenarios.
2. EfficientRepBiPAN, a lightweight network, is employed as the neck network, supplanting the conventional public convolutional layer in the baseline model. This substitution facilitates more flexible and effective feature map information dissemination, utilizing fewer computational resources.
3. We constructed a comprehensive dataset comprising 6846 images of dairy and beef cattle in diverse and complex scenarios based on the publicly accessible dataset created by Fan Q et al.
4. Compared to alternative pose estimation algorithms, our method achieves an improvement of at least 0.4% in AP_0.5_ while maintaining a relatively low parameter count and computational effort.

## 2. Materials

### 2.1 Data acquisition

The data employed for estimating cattle pose branches into two distinct segments: the first encompasses 3934 images of cattle sourced from the publicly accessible dataset by Fan Q et al. Nonetheless, these images exhibit two salient limitations: a paucity of diversity in shooting angles and simplistic backgrounds. These drawbacks may restrict the generalizability and robustness of the resulting model during training. The other portion comprises cattle images captured within the GanTong Dairy Farm in Dali Bai Autonomous Prefecture, Yunnan Province, utilizing an iPhone 12 as the data acquisition device. A total of 167 videos depicting cattle in their natural habitat were recorded. These videos underwent editing and image filtering, yielding 2,912 usable images. Consequently, our dataset consists of a total of 6846 images, which include both dairy and beef cattle with a ratio of approximately 5:1. Each image contains between 1 and 3 cattle, totaling 8600 cattle instances. The dataset is divided into training and validation sets in a 9:1 ratio, with the remaining 1765 images constituting the test set.

**Fig 1:**
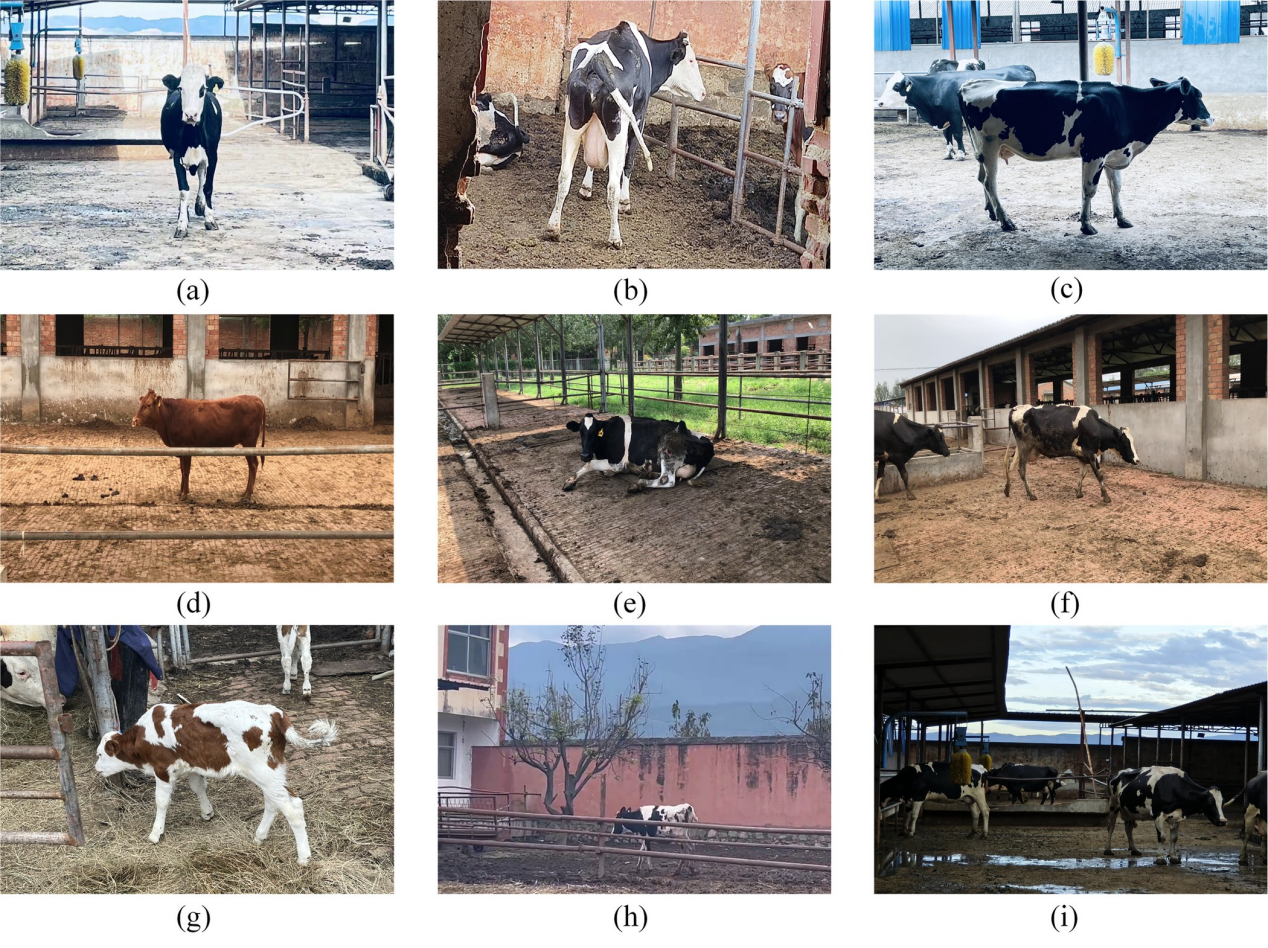
Examples of the supplementary material. (a) Front view. (b) Back view. (c) Cattle blocking. (d) Fence blocking. (e) Lying down. (f) Keypoints missing. (g) Calchood. (h) Remote. (i) Dim light.

The dataset captures various postures of the cattle under different lighting conditions, including standing, walking, lying down, eating feed, and interacting with other cattle. The heterogeneity of these poses, combined with challenges such as occlusion, lighting variances, and camera angles, impacts the precision of cattle pose estimation. This study utilized a range of cattle images encompassing various complex scenes to augment the stability and adaptability of the model. Five primary challenges were identified. First, identifying the positions of keypoints from frontal and rear perspectives is difficult. Second, occlusion issues, including intercattle occlusions, obstructions by fences and posts, and self-occlusion in recumbent poses, may occur. Third, keypoints are missing, particularly when cattle are at the edge of the image, leading to potential loss of vital points. Fourth, keypoints are evident in smaller representations of young or distant cattle. Fifth, distinguishing cattle from similar environmental hues is challenging, especially under dim lighting conditions in farm settings. This issue is crucial for accurately extracting skeletal features. Fig 1 illustrates an example. The training, validation, and test sets all included cattle images from various influencing factors, with similar proportions in the training and validation sets. This dataset stands out from traditional computer vision datasets due to its multiscale, multiscene, and multiangle features, which present a more challenging landscape.

### 2.2 Data preprocessing and labeling

**Fig 2:**
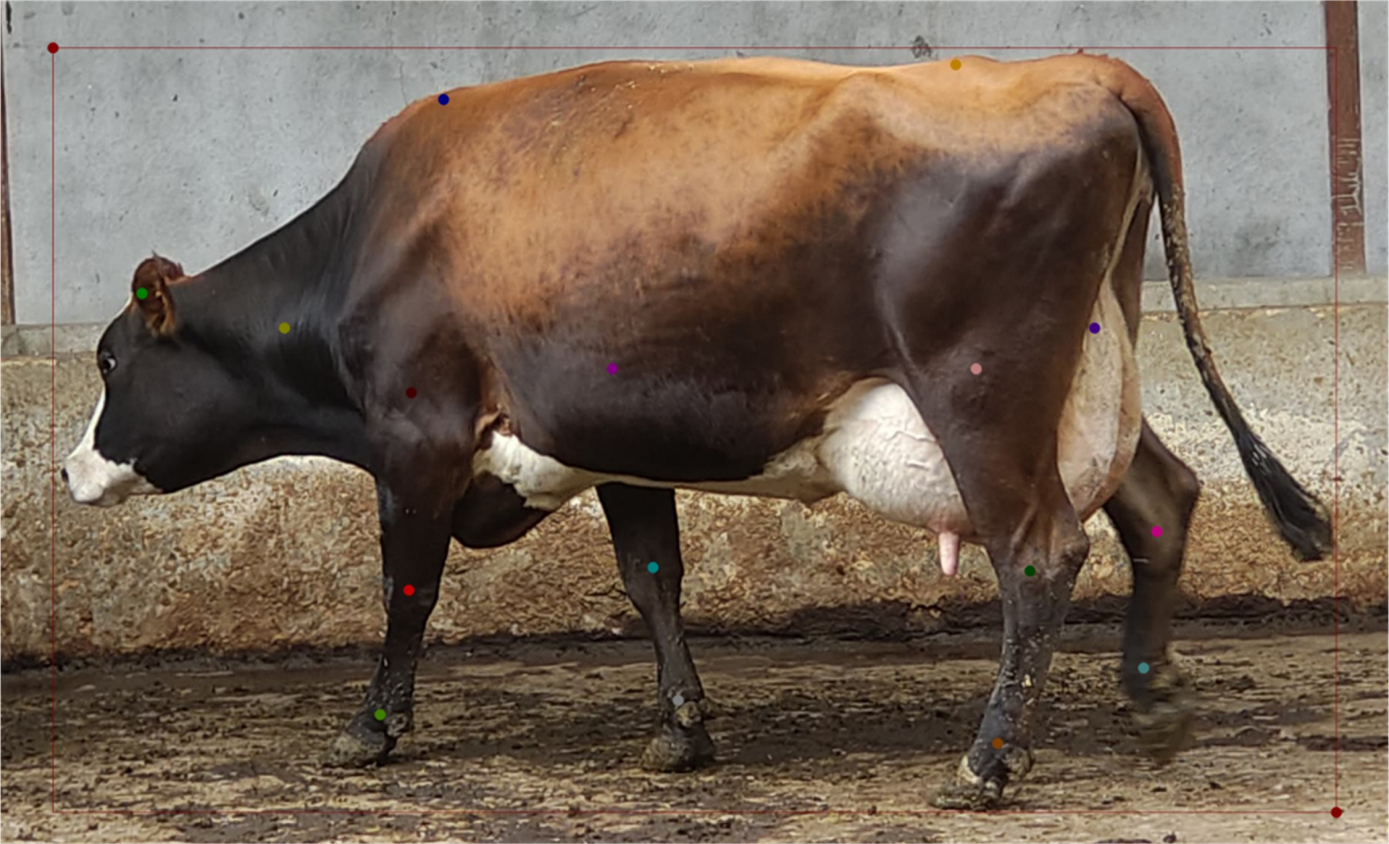
Cattle image labeling.

The novel dataset was annotated using the Labelme tool, which incorporates bounding boxes and keypoints. The bounding boxes cover the entire body of the cattle, while the keypoints are annotated according to Fan Q.’s established research methodology. The sequence of cattle keypoints is as follows: head top, neck, spine, right front thigh root, right front knee, right front hoof, left front thigh root, left front knee, left front hoof, coccyx, right hind thigh root, right hind knee, right hind hoof, left hind thigh root, left hind knee, and left hind hoof. Fig 2 shows the cattle image annotations. All annotated data, including image path, width, height, and number of channels, were archived in the standardized JSON format, and for cattle annotation, the nomenclature and spatial coordinates of the keypoints were used. These JSON files were subsequently transformed into .txt files to meet model training requirements.

## 3. Methods

### 3.1 Standard YOLOv8 model

**Fig 3:**
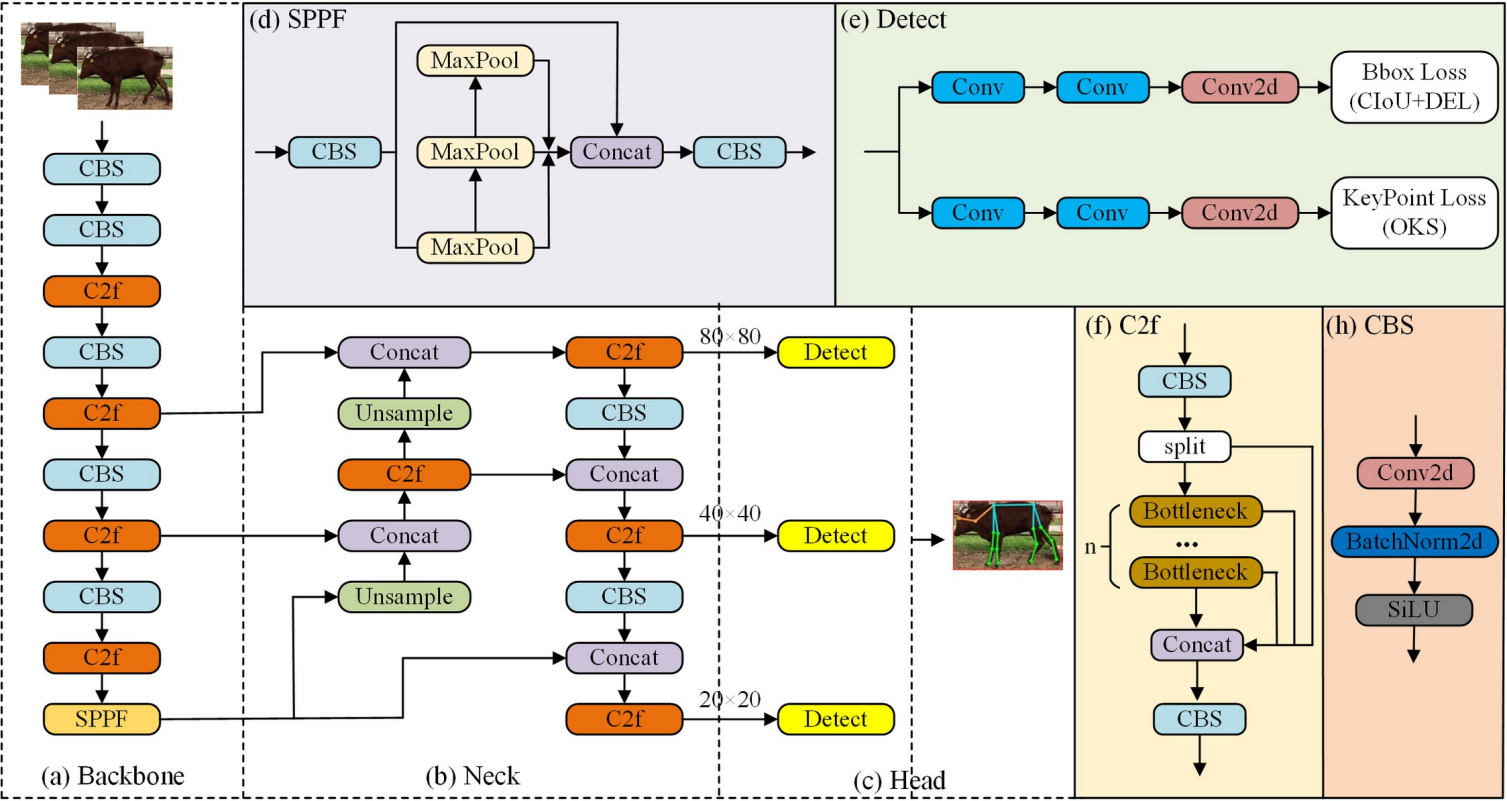
Standard YOLOv8 network structure.

The YOLOv8-pose estimation algorithm is a deep convolutional network based on YOLOv8. It adds to estimating the position of keypoints of the human body pose on the basis of target detection. This algorithm is mainly used for detection and pose estimation tasks involving the human body. To address these diverse requirements, YOLOv8 offers five network versions, varying in depth and width, namely, YOLOv8n, YOLOv8s, YOLOv8m, YOLOv8l, and YOLOv8x. As the accuracy improves, the number of model parameters and computations also increase substantially. This paper focuses on the lightweight YOLOv8n network, which comprises an input, a backbone network, a neck network, and a multiscale prediction output header. The input adapts image scaling for resizing and employs mosaic data augmentation for enhanced robustness. The backbone network extracts features from the input image to capture feature information at various scales and semantic levels. The neck network utilizes a PAN structure to fuse object features at different scales, propagate features at different levels, and improve model detection performance. The multiscale prediction output head employs three groups of detectors of varying sizes to predict image keypoints for target detection. Fig 3 illustrates the standard YOLOv8 network structure.

### 3.2 Improved YOLOv8 cattle pose estimation model

In this work, using the YOLOv8n network with three-branch feature output as a baseline model, a pose estimation algorithm is proposed that incorporates the EfficientRepBiPAN network and SimAM. This algorithm decreases the network architecture, concurrently augmenting the precision of keypoint detection.

**Fig 4:**
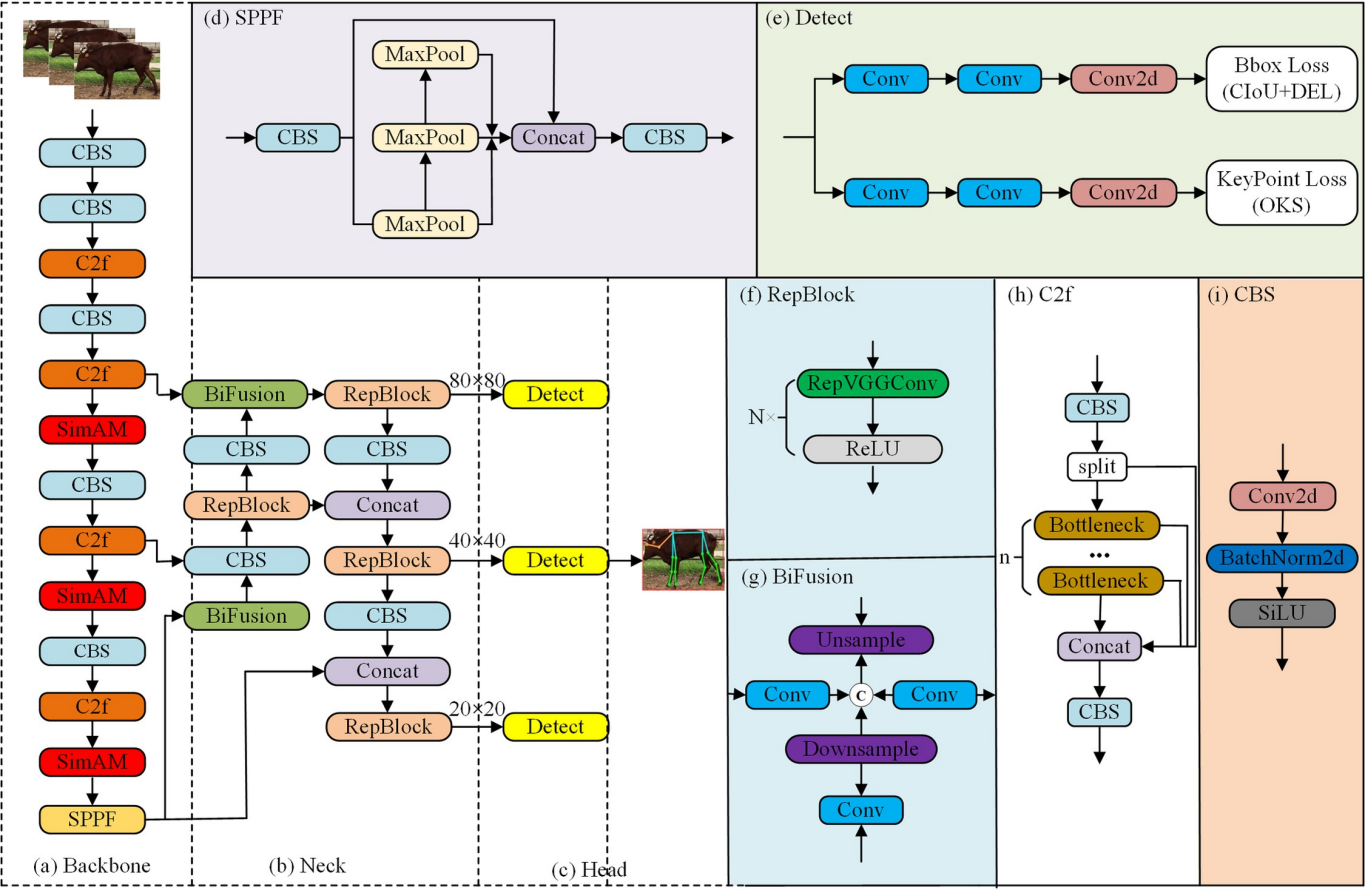
Improved YOLOv8 network architecture.

Specific enhancements entail the integration of the SimAM attention mechanism subsequent to the C2f module within the P3, P4, and P5 layers of the backbone network. This mechanism dynamically adjusts the feature map weights by calculating the similarity and diminishing the training weights of the background features. Consequently, the algorithm concentrates more profoundly on the spatial information of the features, thereby elevating the overall feature extraction performance. In addition, EfficientRepBiPAN is adopted as the neck network and is designed to yield more effective feature information while utilizing fewer computational resources. This approach not only accelerates network detection speed but also alleviates the problem of network redundancy. The architectural schematic of the improved YOLOv8 is shown in Fig 4.

### 3.3 SimAM attention mechanism

**Fig 5:**
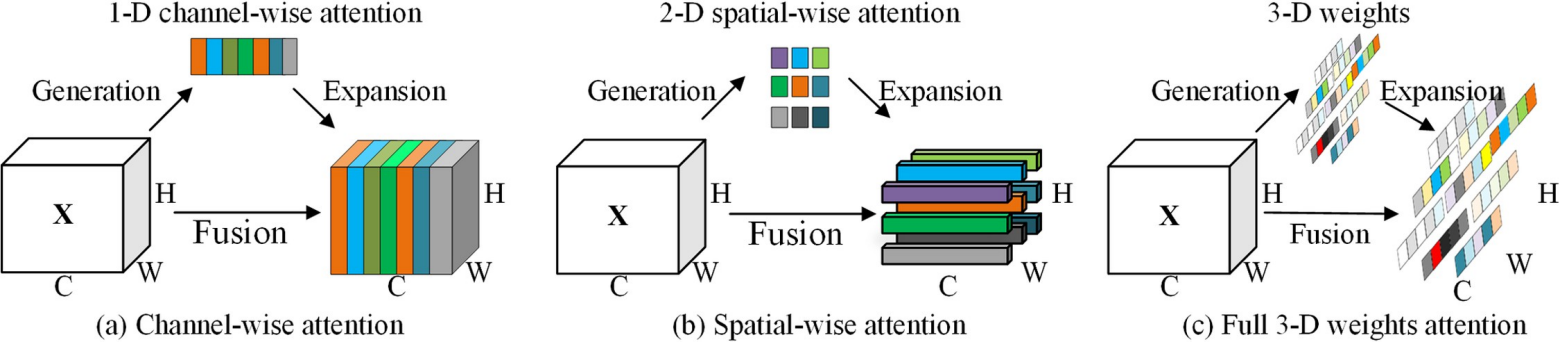
Comparison of the three attention steps.

Existing attention modules typically operate along channel or spatial dimensions, as exemplified in Fig 5A and 5B. These modules generate one-dimensional or two-dimensional weights, treating neurons uniformly across each channel or spatial location and constraining the model’s ability to discriminate between features, correlate features, and flexibly. In contrast, the SimAM attention mechanism combines the channel dimension and the spatial dimension by formulating three-dimensional, unparameterized attention weights that unify channel and spatial scales, as shown in Fig 5C.

According to some well-established neuroscientific theories, information-rich neurons tend to exhibit firing patterns different from those of surrounding neurons. These active neurons exert a substantial spatial inhibitory influence on the activity of peripheral neurons, a phenomenon termed ’null-space inhibition’. Greater importance should be given to neurons with null-space inhibitory effects. The SimAM attention mechanism for finding important neurons: Greater importance should be given to neurons with null-space inhibitory effects. The SimAM attention mechanism for finding important neurons: a measure of the linear separability between neurons. Therefore, the following energy function is defined for each neuron. Therefore, a specific energy function is established for each neuron.

$$e_{t}=\frac{4(\frac{1}{M}\sum_{i=1}^{M}{\left(x_{i}-\varepsilon \right)}^{2}+\omega )}{{\left(t-\varepsilon \right)}^{2}+2(\frac{1}{M}\sum_{i=1}^{M}{\left(x_{i}-\varepsilon \right)}^{2}+\omega )}$$ (1)

$$\varepsilon =\frac{1}{M}\sum_{i=1}^{M}x_{i}$$ (2)

where *t* and *x*_*i*_ represent the input feature $X\in R^{C\times H\times W}$ cattle feature maps of the target neurons and other neurons in a single channel. *i* is an index in the spatial dimension. The number of neurons on that channel can be obtained by *M = H×W*. *ε* denotes the average value calculated for all neurons in the channel except the target neuron *t*. *ω* is the regularization coefficient, typically set to 1e-4. The importance of each neuron is obtained by dividing *1/e*_*t*_. A lower value of *e*_*t*_ indicates that the target neuron *t* in the cattle feature map is distinct from the rest of the neurons. Refine the features using scaling operators; the entire refinement phase of the SimAM module is:

$$\tilde{X}=X⊙sigmoid(\frac{1}{E})$$ (3)

In this formula, where *E* groups all *e*_*t*_ across channels and spatial dimensions, the sigmoid function is added to limit *E* values from being too large. Since the sigmoid function is a single peak function, it does not affect the relative importance of each neuron.

### 3.4 Lightweight Network—EfficientRepBiPAN

**Fig 6:**
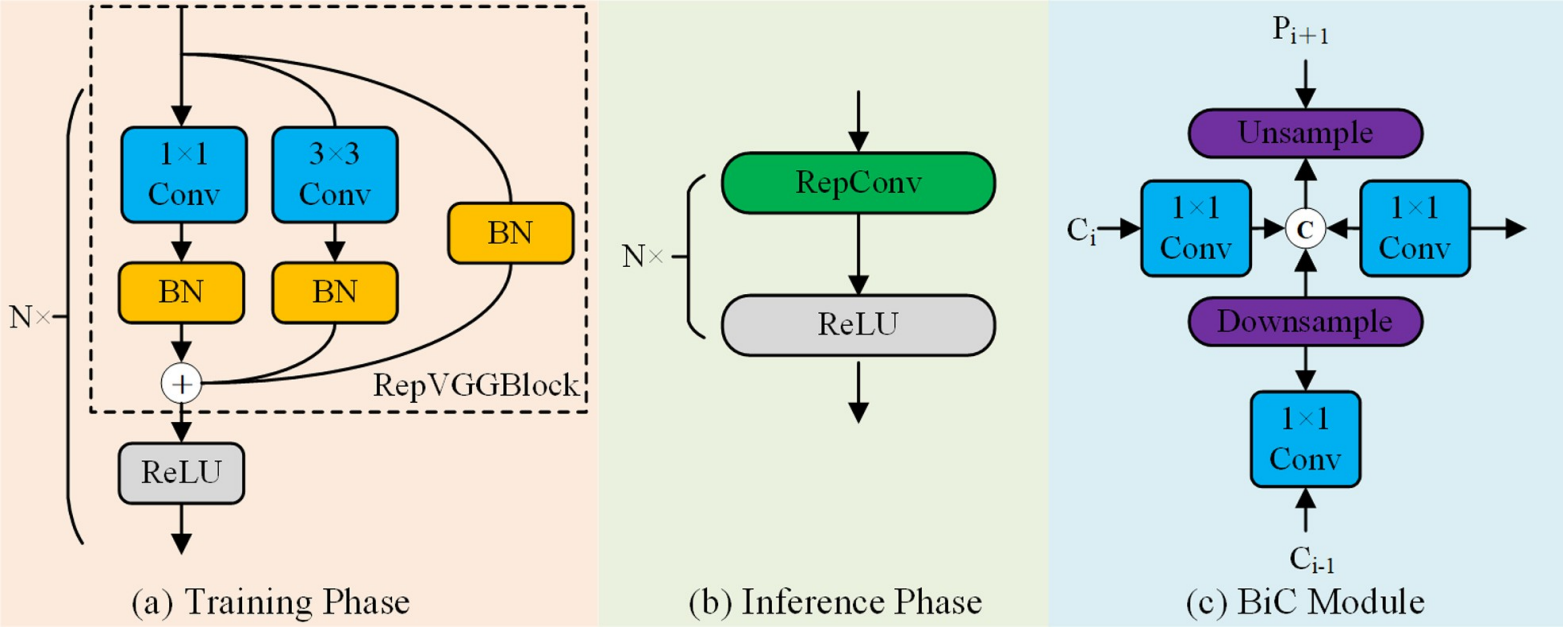
EfficientRepBiPAN network structure.

RepVGG innovatively proposes a structural reparameterization method that uses a multibranch structure in the training phase and converts to a single-branch structure in the inference phase, effectively achieving a balance between speed and accuracy. Inspired by this idea, we adopted the EfficientRepBiPAN architecture based on YOLOv6 [30] when designing the neck network for YOLOv8. The structure uses a reparameterized approach and consists mainly of conv, RepBlock and BiFusion modules. During the training phase, EfficientRepBiPAN implements a complex multibranch structure through RepBlock, as illustrated in Fig 6A. Instead, in the inference phase, each RepBlock is efficiently converted into a single stacked 3×3 convolutional layer accompanied by a ReLU activation function called RepConv, as shown in Fig 6B.

This paper draws on the design concepts of feature pyramid networks (FPNs), bidirectional feature pyramid networks (BiFPNs) and PANet to propagate semantic and location information from bottom-up paths and introduces the bidirectional concatenation (BiC) module, i.e., the BiFusion module. This module centers on the integration of feature maps of the three neighboring layers by efficiently fusing an additional low-level feature from the Ci layer of the backbone to the P_i+1_ layer, as shown in Fig 6C. Although current bidirectional methods consume memory and bandwidth, they are able to predict candidate targets more accurately by creating new feature maps from a shallow level for feature fusion. Due to its residual nature, this new feature pyramid can be easily trained and efficiently integrated into different network structures.

## 4. Experimental results

### 4.1 Experimental environment and training strategies

Table 1 displays the hardware platform and environmental parameters utilized during the experimental training phase.

**Table 1:** Hardware platform and environment parameters.

Parameters	Configuration
CPU	Intel Core i5-13600KF
GPU	NVIDIA RTX 4070 Ti
GPU memory size	16GB
Operating systems	Windows11
Deep learning architecture	Pytorch1.12.1+Cuda11.6+cudnn8.6

Before training the model, setting the hyperparameters has a significant impact on its effectiveness. After conducting several experiments, adjustments were made. The network was optimized using stochastic gradient descent as an optimizer. To ensure the model’s stability, we implement a warm-up strategy during training. This involves using a learning rate of 0.1 for warm-up training within the first 3 rounds, after which the learning rate is restored to the initial learning rate. The initial learning rate is set to 0.01, the final learning rate is set to 0.0001, the weight decay coefficient is set to 0.0005 and the momentum factor is set to 0.937. During the training cycle, a batch size of 32 was used, and the best parameters were saved for evaluating the model’s performance. Mosaic data augmentation was disabled during the final 10 stages of the training process to accelerate the model’s convergence.

### 4.2 Evaluation indicators

The validation standard in our experiment adopted the average accuracy mean metric based on the object key similarity (OKS) validation standard officially given by MS COCO, where *L*_*oks*_ is indicated as follows:

$$L_{oks}=\frac{\sum_{i}\exp(-d_{i}^{2}/2s^{2}k_{i}^{2})\delta (\nu_{i}>0)}{\sum_{i}\delta (\nu_{i}>0)}$$ (4)

$$AP=\frac{M_{d}(L_{oks}\geq t)}{M_{d}}$$ (5)

where *i* is the labeled keypoint number. *d*_*i*_ is the Euclidean distance between the true and predicted keypoints. *s* is the target scale. *k*_*i*_ is the control decay constant at the i^th^ critical point (*sk*_*i*_ is the standard deviation). *v*_*i*_ indicates whether the keypoint of the actual cattle body may be seen (*v*_*i*_ > 0 indicates that the keypoint position can be observed; *v*_*i*_ ≤ 0 indicates that the keypoint position cannot be observed).*δ* is the impulse function,*δ*(*v*_*i*_ > 0) indicates that *L*_*oks*_’s values are calculated solely for visible keypoints in the actual annotation, as indicated. The possible values for *L*_*oks*_ range from 0 to 1. The values of *L*_*oks*_ approach 0 as the difference between the true and predicted keypoints increases and approach 1 as the two points become infinitely close.

*L*_*oks*_ are analogous to intersection-to-union (IoU) ratios in target detection and are used to compute the AP of keypoint detection. AP represents the average detection accuracy when *L*_*oks*_ takes values between 0.50 and 0.95 with a step size of 0.05. AP_0.5_ represents the detection accuracy when *L*_*oks*_ = 0.50, which is utilized for the validation assessment of test set results. Furthermore, this study proposes SimAM-EfficientRepBiPAN, a lightweight model evaluated based on important metrics such as floating-point operations (FLOPs) and the number of parameters (Parameters). The floating-point arithmetic volume is calculated by multiplying the number of parameters in each layer by the dimension of the input data and then summing the results across all layers. The number of parameters represents the number of parameters in the model and is often used as a measure of the complexity and capacity of the model.

### 4.3 Comparative experiments of various attention mechanisms

This study uses the YOLOv8n-pose estimation network as the baseline model, introducing attention mechanisms such as MLCA (Multi-Level Cross Attention) [31], CAFM (Channel Attention Feature Module) [32], MPCA (Multi-Path Channel Attention) [33], and SimAM into the original backbone network. By maintaining the parameters outside the backbone network consistent, the experimental results in the table demonstrate the performance of the backbone network incorporating various attention mechanisms on the test set. The results in Table 2 depict that the model with the SimAM attention mechanism outperforms others, improving AP_0.5_ by 1.3%, 3.8%, and 1.7%, and AP_0.5:0.95_ by 1.5%, 10.1%, and 5.8%. Therefore, the SimAM attention mechanism enhances the backbone network’s feature extraction capability and improves the detection performance of the YOLOv8n-pose estimation model.

**Table 2:** Comparison of various attention mechanisms in backbone networks.

Baseline Model	Attention mechanisms	AP_0.5_	AP_0.5:0.95_	Network Layers	Model Size(MB)
YOLOv8n	MLCA	90.3%	50.0%	202	7.5
YOLOv8n	CAFM	87.8%	41.4%	205	8.5
YOLOv8n	MPCA	89.9%	45.7%	220	8.4
YOLOv8n	SimAM	91.6%	51.5%	273	6.6

### 4.4 Comparative experiments with different neck network feature fusion structures

Four distinct feature fusion architectures—AFPN (Adaptive Feature Pyramid Network) [34], FDPN (Feature Decomposition Pyramid Network) [35], HSFPN (High-Scale Feature Pyramid Network) [36], and EfficientRepBiPAN (Efficient Representative Bidirectional Pyramid Aggregation Network)—were integrated into the YOLOv8 neck network, and their performance was evaluated on the test set, with results presented in Table 3.

**Table 3:** Comparison of different feature fusion structures in neural network necks.

Baseline Model	Neck Network	AP_0.5_	AP_0.5:0.95_	Network Layers	Model Size(MB)
YOLOv8n	AFPN	89.9%	44.9%	133	4.8
YOLOv8n	FDPN	91.9%	48.6%	225	7.0
YOLOv8n	HSFPN	90.3%	45.6%	199	4.5
YOLOv8n	EfficientRepBiPAN	91.7%	54.8%	237	6.0

The results reveal that while FDPN achieved an AP_0.5_ of 91.9%, the EfficientRepBiPAN demonstrated superior combined performance in both AP_0.5_ and AP_0.5:0.95_, outperforming AFPN, HSFPN, and FDPN. Despite having the largest total number of network layers, EfficientRepBiPAN maintains a moderate model size. These findings underscore the enhanced efficacy of the EfficientRepBiPAN feature fusion module within the neck network. Consequently, the EfficientRepBiPAN module was selected to optimize the neck network’s performance.

### 4.5 Ablation study on the model performance

This study conducted four sets of ablation experiments to assess the impact of each improvement module on the model’s overall performance. The same equipment and dataset were used for training and testing to ensure the comparability of the results. The experimental results are presented in Table 4. The four sets of experiments included the original baseline models YOLOv8n, YOLOv8n and SimAM; YOLOv8n and EfficientRepBiPAN; and the proposed integrated approach.

**Table 4:** Ablation experiment configurations and results (√ and ‐ denote "with" and "without" the module, respectively).

No.	Baseline	SimAM	EfficientRepBiPAN	AP_0.5_	AP_0.5:0.95_	parameters (M)	FLOPs (G)
1	√	-	-	88.0%	47.4%	3.27	9.1
2	√	√	-	91.6%	51.5%	3.27	9.1
3	√	-	√	91.7%	54.8%	3.11	8.1
**4**	**√**	**√**	**√**	**92.3%**	**56.4%**	**3.11**	**8.1**

**Fig 7:**
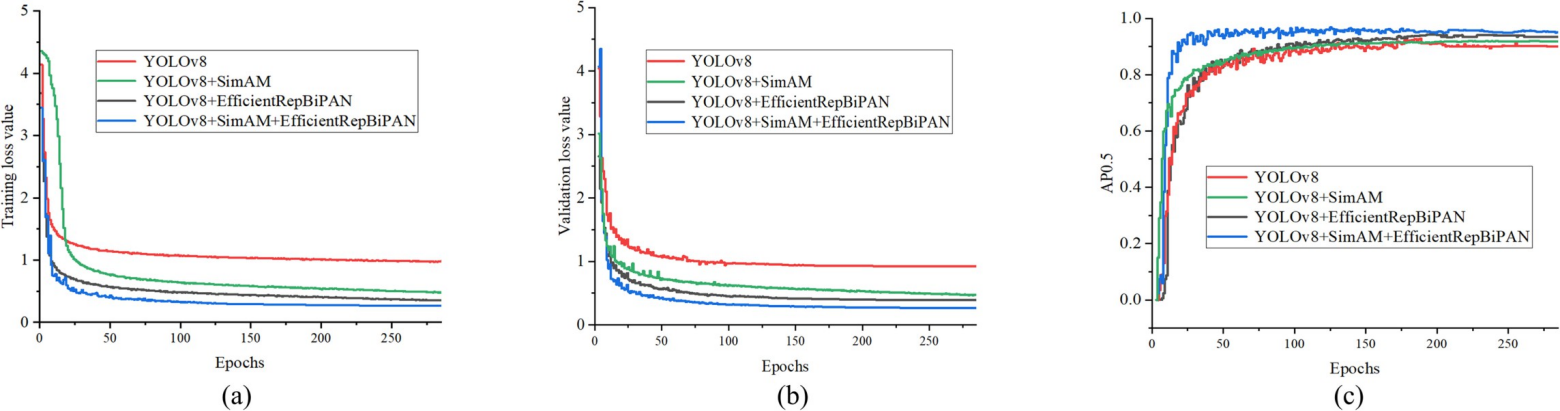
Loss curves and AP_0.5_ curves of models with YOLOv8 as the baseline in each epoch for cattle pose estimation. (a) Training loss. (b) Val loss. (c) AP_0.5_ curve.

Among the training epochs, our focus is on saving the optimal parameters for evaluating the model performance. Fig 7 shows the training loss curves, validation loss curves and AP_0.5_ metric curves for the pose estimation task based on the four model sets with YOLOv8 as the baseline. In Fig 7, it is evident that both the training loss and validation loss curves start to stabilize after approximately the initial 25 training cycles. After approximately 50 training cycles, the AP_0.5_ metric reaches a state of stability. Our method outperforms the other three groups of models in terms of training speed and quality.

The data analysis illustrated in Table 4 elucidates that various enhancement strategies, when integrated into the benchmark model, yield improvements in detection performance to differing extents. The efficient attention mechanism of SimAM augments the focus on pivotal information within the feature map. This enhancement is achieved without escalating the model’s parameter count or computational demand, culminating in a notable 4.1% enhancement of the AP_0.5:0.95_ metric, alongside a marginal increase in detection speed. The EfficientRepBiPAN module not only features a relatively simple structure, but also broadens the model’s receptive field by integrating features from various hierarchical levels, allowing the model to capture target features across different scales and thus enhancing its detection and recognition capabilities in pose estimation tasks. his simplicity does not compromise its effectiveness, as demonstrated by an improvement of 7.4% in the AP_0.5:0.95_ metric. In the integrated implementation of these two modules, the model blends feature layers that are rich in detail, enabling a comprehensive integration of both shallow and deep information. This approach further reduces the leakage rate of cattle targets. In detail, the feature fusion network we propose effectively improves the detection performance of the model while reducing the computational cost.

### 4.6 Comparison of the results on YOLOv8

In the present research, Table 5 delineates the comparative performance of our novel pose estimation method against contemporary state-of-the-art algorithms, as evaluated on our uniquely developed cattle dataset. This analysis underscores the effectiveness and advancements brought forth by our proposed methodology. The results reveal that our proposed network architecture achieves a decrease of 0.16 M in the number of parameters and a notable reduction of 1.0 GFLOPs in computational cost with the input dataset size maintained at 640 × 640 pixels when benchmarked against the original YOLOv8n-pose baseline model. The methodology yields significant enhancements in performance metrics, achieving improvements of 4.3% for AP_0.5_ and 9.0% for AP_0.5:0.95_.

**Table 5:** Comparison of the accuracies of different methods. (The bold data in the table indicate the best results).

Methods	Backbone	Input Size	AP_0.5_	AP_0.5:0.95_	parameters (M)	FLOPs (G)
HRNet	HRNet-W32	256×192	71.9%	37.3%	28.50	16.00
HigherHRNet	HRNet-W32	512×512	71.6%	48.3%	28.65	46.58
DEKR	HRNet-W32	256×192	71.1%	49.9%	29.56	44.62
DEKRV2	HRNet-W32	512×512	75.0%	53.2%	35.34	46.31
YOLOv5s	CSPDarknet-53	640×640	91.9%	56.3%	7.18	16.70
YOLOv7	E-ELAN	640×640	96.3%	81.3%	80.16	101.50
YOLOv8n	CSPDarknet-53	640×640	88.0%	47.4%	3.27	9.10
Ours	CSPDarknet-53	640×640	92.3%	56.4%	3.11	8.10

In contrast to large-scale, high-resolution pose estimation networks such as HigherHRNet and DEKR, our method yields a decrease in computational demand of approximately fivefold in cost and ninefold in parameter count. Moreover, the AP_0.5_ metric increased by 20.7% and 21.2%, respectively. The DEKRv2 algorithm enhances the AP_0.5:0.95_ metric by 1.6% over its predecessor, the DEKR algorithm, while the computational and parameter counts improve. Although HRNet excels in computational cost efficiency, it ranks lowest in the performance metric, AP_0.5:0.95_, compared to other algorithms built on the HRNet-W32 backbone network. Among the YOLO series of pose estimation algorithms, the YOLOv5s-pose and our enhanced YOLOv8n-pose model exhibit comparable performance. YOLOv7-pose stands out with the highest keypoint detection accuracy, achieving an AP_0.5_ of 96.3% and an AP_0.5:0.95_ of 81.3%. However, this superior accuracy is accompanied by substantially higher parameter counts and computational demands. Conversely, our proposed method, while not matching the accuracy of YOLOv7-pose, significantly reduces computational cost and still delivers excellent overall performance

### 4.7 Validation across multiple datasets

This study endeavors to elucidate and substantiate the adaptability and resilience of the refined YOLOv8 algorithm across diverse datasets. Toward this objective, two openly accessible datasets serve as the cornerstone of our experimentation: one sourced from CSDN’s online repository and another contributed by Gong et al. For clear differentiation, we designate the former as ’Farm Cattle’ and the latter as ’Dairy Cattle’. Notably, the Farm Cattle dataset can be promptly retrieved via https://download.csdn.net/download/weixin_43427721/14920332. Precisely, Farm Cattle comprises 327 dairy and beef cattle images, whereas the Dairy Cattle dataset boasts a more ample collection of 1800 dairy cattle images. Each image is included in the validation dataset, with the goal to comprehensively evaluate the performance of the improved YOLOv8 algorithm in pose estimation tasks.

Table 6 demonstrates the model’s performance across multiple datasets both pre- and post-improvement. Compared to its pre-improvement counterpart, the enhanced model exhibits an advancement of 4.1% in AP_0.5_ for the Farm Cattle dataset, alongside a 3.9% enhancement in AP_0.5:0.95_. Similarly, for the Dairy Cattle dataset, AP_0.5_ experiences an uptick of 1.6%, while AP_0.5:0.95_ sees a 4.0% improvement. These experimental results not only fully validate the effectiveness of our proposed method, but also underscore its superior generalization ability and robustness.

**Table 6:** Comparative of YOLOv8n and our model on multiple datasets.

Validation datasets	Quantities	Model	AP_0.5_	AP_0.5:0.95_
Farm Cattle	327	YOLOv8n	83.3%	32.0%
		Ours	87.4%	35.9%
Diary Cattle	1800	YOLOv8n	95.5%	43.1%
		Ours	97.1%	47.1%

### 4.8 Visualization and discussion

In a farm setting, the accuracy of cattle stance estimation can be affected by various environmental factors. For instance, cattle exhibit distinct characteristics under varying lighting conditions. This complexity is amplified by the fact that cattle are categorized into two growth stages, juvenile and adult, each with notable differences in body shape and size. Moreover, the camera captures images of the cattle from multiple angles as they move, including frontal views showcasing full features, side views revealing partial features, and rear views with limited feature visibility. Occlusions limited feature visibility, as did occlusions caused by fences, other cattle, people, or animals. The efficacy of the YOLOv8-pose model was evaluated for estimating cattle poses under these conditions. A specific percentage of images was selected from the total dataset. Table 7 illustrates the details of this extracted dataset.

**Table 7:** Information on the extracted dataset.

Influence factors	Number of images (sheets)
Large scale	200
Small scale	100
Bright light	150
Dim light	150
Front view	100
Side view	100
Back view	100
Occlusion	100
Unobstructed	200

**Fig 8:**
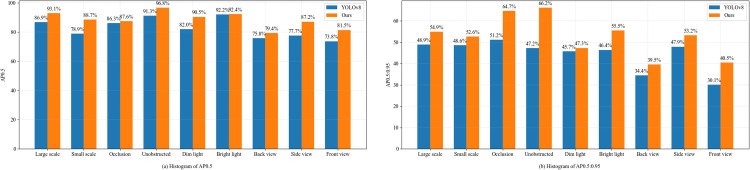
Detection effect of the model before and after improvement in various complex environments. (a) Histogram of AP_0.5_. (b) Histogram of AP_0.5:0.95_.

Fig 8 offers a detailed comparative analysis of the enhanced cattle pose estimation model, illustrating its performance under a range of conditions, including different body sizes, varying levels of illumination, and multiple photographic angles. In instances involving cattle of diverse sizes, the original YOLOv8-pose model demonstrated a difference of approximately 8.0% in AP_0.5_ detection performance when contrasted with its enhanced counterpart. With the refined model, an 88.7% improvement in AP_0.5_ detection accuracy was attained, even when the model was applied to cattle with smaller body scales. For various photographic angles, the model demonstrated its highest efficacy in side pose estimation prior to improvement, achieving an AP_0.5_ of 77.7%. However, this approach was less effective at detecting frontal poses, primarily due to occlusion of key parts by the objects themselves, resulting in a lower AP_0.5_ of 73.8%. The enhanced YOLOv8 model achieved an increase in AP_0.5_ of 7.7% in the frontal position and 3.6% in the dorsal position. In scenarios with occlusion, the AP_0.5_ for the improved model reached 87.6%, demonstrating that the enhanced YOLOv8-pose model is more precise than its predecessor in estimating a cattle’s pose under occlusion conditions.

Fig 8 illustrates that both the advanced YOLOv8-pose model and the original version can precisely determine the cattle’s pose under conditions of stronger light. Under low light conditions, where the background is dimmer, the cattle color merges with the environment, causing their features to become less distinct and blur. However, the improvement in the YOLOv8-pose model at AP_0.5_ was 8.5% greater than that in the initial model. To summarize, under various influencing factors, such as occlusion, lighting, body size, and angle, the enhanced YOLOv8-pose model showed at least a 2.6 percentage point improvement in AP_0.5:0.95_ compared to the original model. This advancement offers crucial informational support for studies in cattle behavioral recognition and related fields.

**Fig 9:**
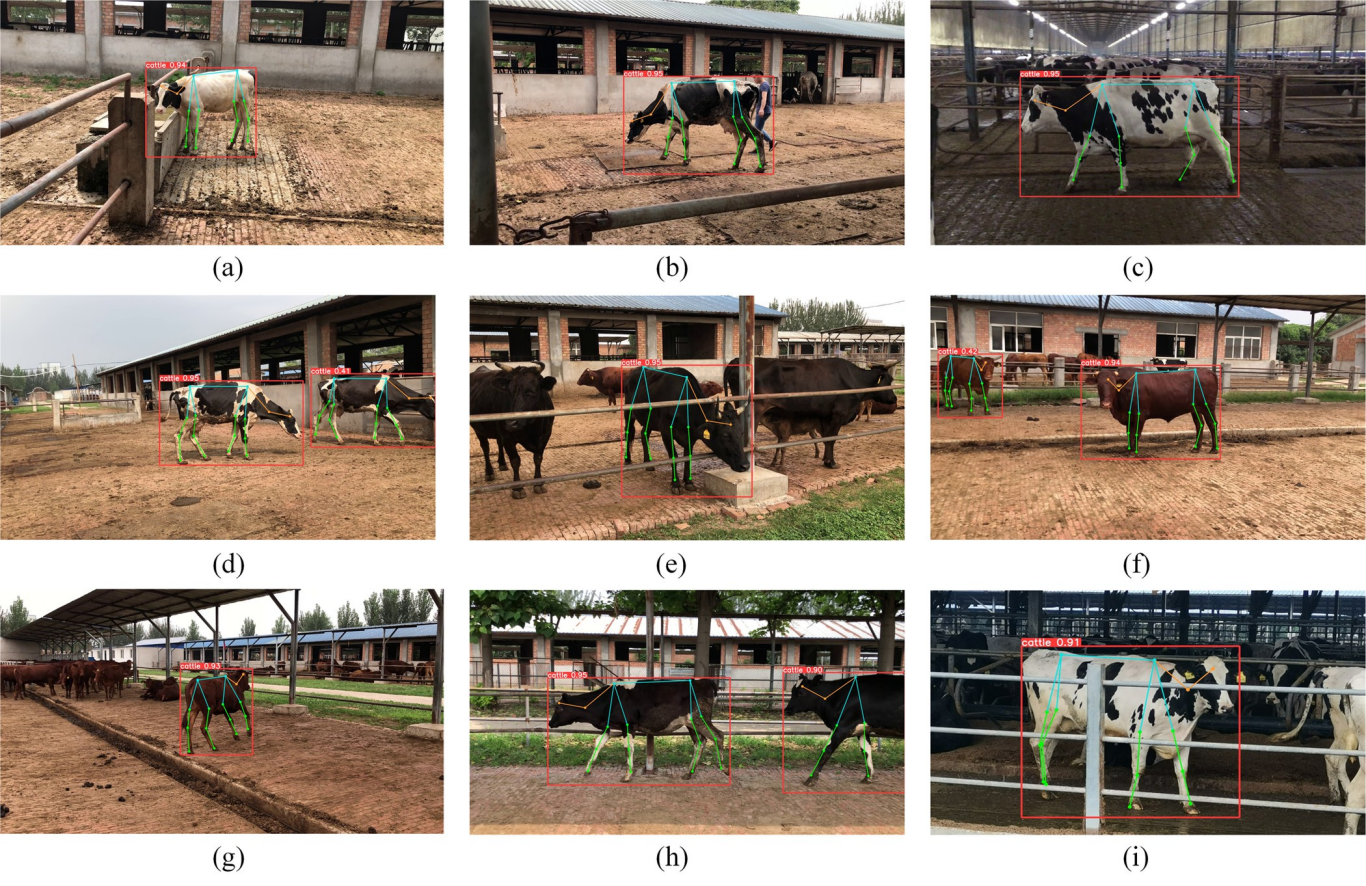
Group of images illustrating the results of improving the YOLOv8-pose model in complex environments involving multiscale objects, multiangle objects, and light intensity problems. (a) Small scale. (b) Large scale. (c) Dim light. (d) Bright light. (e) Front view. (f) Side view. (g) Rear view. (h) Keypoints missing. (i) Fence blocking.

Fig 9A and 9B showcase the versatile generalization capability of the optimized YOLOv8-pose model for cattle of varying sizes, accurately predicting their pose regardless of substantial size differences. Despite the challenges posed by fluctuating light intensities throughout the day and the difficulty in distinctly discerning the cattle’s silhouette under certain barn conditions, the model excels in capturing complete positional details, as evidenced in Fig 9C and 9D. Fig 9E–9G illustrate the model’s ability to estimate cattle poses from diverse perspectives: frontal, lateral, and rear. The model consistently exhibited stable recognition of cattle poses from these varied viewpoints. Furthermore, Fig 9H highlights the model’s capacity to precisely assess the cattle’s stance, even in the absence of critical body parts. Fig 9I demonstrates effective pose estimation when the cattle are partially concealed by barn fencing, affirming the model’s robust performance in complex settings.

## 5. Conclusion

Conventional methods for the manual observation of animal behavior are notably inefficient, demanding substantial time and workforce, whilst being susceptible to the biases inherent to human observers. Alternatively, the deployment of wearable sensors for behavior monitoring, despite offering valuable insights, harbors the potential to elevate disease risk amongst the studied fauna. In contrast, deep learning-based technologies markedly surpass these traditional approaches, offering significant advantages in terms of time and labor economization, minimization of adverse welfare implications for animals, and the facilitation of analyses that are both more objective and precise in their behavioral assessments.

In addressing the challenge of estimating cattle poses, this study introduces an end-to-end YOLOv8-pose method, which provides valuable information for animal behavior studies and can further support gait analysis [37], health status assessment, and oestrus detection in livestock farming. Experimental data show that the proposed method achieves a 92.3% on the AP_0.5_ metric, representing a considerable improvement over its baseline model. This strategic decision not only substantially reduces the computational complexity and parameter size of the model but also streamlines the architecture, notably enhancing detection speed. Such enhancement is consistently reflected across all the performance indicators. The integrated network effectively handles the task of estimating cattle poses across diverse body sizes, various shooting angles, complex lighting scenarios, and different degrees of occlusion. The improved model achieves an enhancement of at least 2.6 percentage points in the AP_0.5:0.95_ metric. However, the method still has limitations in detecting animals that tend to cluster together. Future efforts will be directed toward developing a more comprehensive dataset for cattle pose estimation to enhance the model’s performance and adaptability. Therefore, we expect to further improve the accuracy of the cattle pose estimation model and contribute to animal pose estimation in livestock production.
